# The Roll of Emetine in the Treatment of Pyorrhea Alveolaris*Read before the Chattahoochee Valley Medical and Surgical Association, Opelika, Ala., July 15, 1915.

**Published:** 1915-07

**Authors:** William Crenshaw

**Affiliations:** Dean, Atlanta Dental College; Dental Surgeon to Grady Hospital 619-20-21 Grant Bldg., Atlanta, Ga.


### THE ROLE OF EMETINE IN THE TREATMENT OF PYORRHEA ALVEOLARIS*

By William Crenshaw D. D. S., Dean, Atlanta Dental College.

Dental Surgeon to Grady Hospital.

Since the discovery of the ameba buccalis simultaneously by Chiavaro in Italy and Smith and Barrett in this country, much has been said about the relation of this parasite to pyorrhea alveolaris. The amebae may easily be demonstrated in the exudate from the pyorrhetic pockets about the teeth in cases of Riggs' disease; and it is possible that these parasites aid in the maintenance and spread of the suppurative process. Bass and Johns believe that the amebae in their capacity of scavengers for disposing of bacterial refuse act as carriers for pathogenic organisms, that the motility of the amebae make them vehicles for spreading infections into the healthy tissue adjacent to the suppurating area. Nevertheless we should bear in mind that the function of amebae is beneficient rather than pathogenic, and that whatever damage they do as germ carriers is incidental.

It is doubtful whether the killing and elimination of amebae from pyorrhea pockets accomplishes anything toward putting a stop to suppuration. So long as pathogenic organisms are left in full sway the absence of amebae will not benefit the patient. the patient.

If, however, steps are taken to sterilize the pyorrhetic pockets, then the elimination of germ carrying amebae becomes an aid toward cure. Dr. Geo. B. Clement, of Meridian, Mississippi, whom I regard the foremost authority on Riggs' disease in this country, claims that amebae are attracted to pyorrhea pockets because of the peculiar pabulum created as a byproduct, so to speak, of the work of bacteria; acordingly the amebae are found in pyorrhea pockets as a result of the pyorrhetic condition and not as the cause.

Emetine hydrochloride administered hypodermically in half grain doses for several days—say six or eight days, saturates the blood with the alkaloid and kills the amebae found in pyorrhea pockets. Applied locally in one-half or one per cent solution, this alkaloid also proves fatal to the parasites. Emetine, acts destructively upon the amebae, and is probably made efficacious acting through the blood, because in this way it more thoroughly reaches all of the parasites than could be done by local applications. It should not be forgotten in this connection that emetine arrests bleeding at the line of demarcation in suppurative pockets, not because it kills amebae, which it does, but by virtue of its hemostatic potency. This damming back of blood and pus by coagulation is mistaken for cure; and explains why differental diagnosis between early pulmonary tuberculosis and chronic bronchitis. These two are often confused by one not and how emetine in the hands of the uninitiated seems to be curing the disease.

Other agents such as tincture of iodine and silver nitrate solution, twenty grains to the ounce, will destroy the amebae in Riggs' disease by local application, and these germicides have the further advantage of destroying the pathogenic organisms such as staphylococci, streptococci, pneumococci, etc. In such, however, as the amebae buccalis ordinarily found in the saliva of the mouth, neither emetine nor iodine nor silver nitrate will do more than temporarily eliminate these parasits. Even after a six-day series of emetine injections the amebae will re-appear in the course of time. Likewise, pathogenic germs will come again as the buccal cavity is a never failing source of these organisms; and we cannot, therefore, hope to cure Riggs' disease merely by the use of agents for the destruction of parasites and bacteria.

Nothing short of thorough surgical work with properly adapted instruments, and the use of appropriate germicidal measures will surely and permanently arrest the stubborn malady known as pyorrhea alveolaris. The role of emetine is secondary, though helpful, the same being true of both tincture of iodine and silver nitrate solution.

Preferably the writer gives each pyorrhea patient the benefit of a six-day series of emetine injections, one-half grain ampule at a dose, and also makes free use of iodine and silver nitrate locally, but depends upon surgical removal of irritating calculi from the roots of the teeth and walls of the alveoli for permanent curative results.

In conclusion, it should be remembered that the injection of emetine is more or less painful when done subcutaneously; but is rendered painless and much more satisfactory when done, as suggested by Dr. E. C. Thrash, of Atlanta, intravenously with an ordinary hypodermic syringe.

619-20-21 Grant Bldg., Alanta, Ga.

---

*Read before the Chattahoochee Valley Medical and Surgical Association, Opelika, Ala., July 15, 1915.

